# Emergent Biomarkers of Residual Cardiovascular Risk in Patients with Low HDL-c and/or High Triglycerides and Average LDL-c Concentrations: Focus on HDL Subpopulations, Oxidized LDL, Adiponectin, and Uric Acid

**DOI:** 10.1155/2013/387849

**Published:** 2013-11-04

**Authors:** Filipa Mascarenhas-Melo, Filipe Palavra, Daniela Marado, José Sereno, Edite Teixeira-Lemos, Isabel Freitas, Maria Isabel-Mendonça, Rui Pinto, Frederico Teixeira, Flávio Reis

**Affiliations:** ^1^Laboratory of Pharmacology & Experimental Therapeutics, IBILI, Faculty of Medicine, Sub-Unit 1 (Pólo III), University of Coimbra, 3000-548 Coimbra, Portugal; ^2^Neurology Department, General Hospital, University and Hospital Centre of Coimbra, Quinta dos Vales, São Martinho do Bispo, 3041-801 Coimbra, Portugal; ^3^Internal Medicine Department, General Hospital, University and Hospital Centre of Coimbra, Quinta dos Vales, São Martinho do Bispo, 3041-801 Coimbra, Portugal; ^4^ESAV, Polytechnic Institute of Viseu, 3504-510 Viseu, Portugal; ^5^Research Unit, Central Hospital of Funchal, 9004-514 Funchal, Madeira, Portugal; ^6^Laboratory of Human Genetics, Campus Universitário da Penteada, 9020-105 Funchal, Madeira, Portugal; ^7^Pharmacology and Pharmacotoxicology Unit, Faculty of Pharmacy, University of Lisbon, 1649-003 Lisbon, Portugal

## Abstract

This study intended to determine the impact of HDL-c and/or TGs levels on patients with average LDL-c concentration, focusing on lipidic, oxidative, inflammatory, and angiogenic profiles. Patients with cardiovascular risk factors (*n* = 169) were divided into 4 subgroups, combining normal and low HDL-c with normal and high TGs patients. The following data was analyzed: BP, BMI, waist circumference and serum glucose, Total-c, TGs, LDL-c, oxidized-LDL, total HDL-c and HDL subpopulations, paraoxonase-1 (PON1) activity, hsCRP, uric acid, TNF-**α**, adiponectin, VEGF, and iCAM1. The two populations with increased TGs levels, regardless of the normal or low HDL-c, presented obesity and higher waist circumference, Total-c, LDL-c, Ox-LDL, and uric acid. Adiponectin concentration was significantly lower and VEGF was higher in the population with cumulative low values of HDL-c and high values of TGs, while HDL quality was reduced in the populations with impaired values of HDL-c and/or TGs, viewed by reduced large and increased small HDL subfractions. In conclusion, in a population with cardiovascular risk factors, low HDL-c and/or high TGs concentrations seem to be associated with a poor cardiometabolic profile, despite average LDL-c levels. This condition, often called residual risk, is better evidenced by using both traditional and nontraditional CV biomarkers, including large and small HDL subfractions, Ox-LDL, adiponectin, VEGF, and uric acid.

## 1. Introduction

Dyslipidemia is recognized as one of the major risk factors for the development of cardiovascular disease (CVD), which remains the leading cause of death across Europe [1]. The 3-hydroxy-3-methylglutaryl-coenzyme (HMG-CoA) reductase inhibitors, also known as statins, are the first-line lipid-lowering agents for CVD patients and have transformed the treatment of dyslipidaemia. Effective antidyslipidemic treatment significantly reduces cardiovascular risk: a 10% reduction in total cholesterol is associated with a 25% reduction in the incidence of coronary artery disease (CAD) at five years, and reducing low density lipoprotein cholesterol (LDL-c) by 40 mg/dL (1 mmol/L) with statin therapy leads to a 20% reduction in risk for coronary events [2, 3]; for every mmol/L reduction in LDL-c, the risk of coronary heart disease (CHD) mortality decreases by 19% and overall mortality decreases by 12% [2, 4]. However, a significant number of patients on statin therapy have persistent dyslipidaemia, as demonstrated by the dyslipidemia international study (DYSIS) which reported data from several European countries, including from Portugal [5–8]. 

Recently, several authors have been focusing the attention on the concept of residual cardiovascular risk (RCVR) [9, 10]. According to Hermans and Fruchart, RCVR could be defined by the “residual risk of incident vascular events or progression of established vascular damage persisting in patients treated with current evidence-based recommended care, including risk from established risk factors, such as dyslipidemia, high blood pressure, hyperglycemia, inflammation and unhealthy lifestyles, and risk related to emerging or newer risk factors” [10]. It is apparent that a greater number of residual cardiovascular events occur than are prevented with statin therapy; indeed, the RCVR remains elevated even in clinical trials in which LDL-c levels have been aggressively reduced [11–13]. As a result, there has been an increased focus on elevated triglycerides (TGs) and decreased high density lipoprotein cholesterol (HDL-c) levels and their significant contributions to RCVR even when LDL-c levels are well controlled [14, 15]. 

Low levels of HDL-c have been largely recognized as a risk factor for CHD and high levels as a protective factor, according to epidemiology studies performed in subjects and/or patients not optimally treated with statins [16–18]. Although HDL-c has been traditionally associated to atheroprotection, the concept of “HDL-c quality” as an important parameter in reducing cardiovascular mortality is constantly gaining ground and HDL particle functionality has been recognized as a putative pharmacological target for HDL-based therapies [19–22]. HDL quality refers to the composition and functions of HDL particle subpopulations present in a single individual and may define whether HDL is atheroprotective or even proatherogenic [19, 20]. It has been suggested that monitoring the type of HDL particles (carry distinct and specific proteins or lipids and differentiated by their density and size: large, intermediate, and small), rather than their total quantity, is a more reasonable way of determining the CV risk, suggesting that different subpopulations may have a different role in reverse cholesterol transport (RCT) and CVD risk protection [23, 24]. However, defining HDL quality remains vague and further studies are needed to clarify the true differences between the particles of HDL and to justify their different functionalities. Nevertheless, variations in HDL subfraction levels and functions have been observed in distinct CVD populations, suggesting that large HDL particles are inversely associated with atherosclerosis development while small HDL subpopulations are positively connected with CVD [20–27].

On the other hand, some data suggest that hypertriglyceridemia, as the result of TG-rich lipoproteins overproduction and/or decreased catabolism, is a major factor associated with lack of goals attainment [28]. Elevated TGs levels are considered an independent risk factor for CVD even when controlling for the other factors [29–31], and treatment of elevated TGs in clinical trials has been shown to reduce CVD events, cardiac deaths, and total mortality [31–33]. Patients with elevated TGs are at particularly high risk of CVD, particularly when coexisting with low HDL-c levels [34, 35]. Due to the importance that the levels of HDL-c and TGs have been gaining, the ratio TGs/HDL-c may become a new relevant marker for the determination of cardiovascular risk. In adults, the simple TGs/HDL-c index was shown to identify patients with dyslipidemia and insulin resistance [36, 37]; in overweight adults, Barter et al. [38] recently showed that only those subjects with an increase in TGs and a decrease in HDL-c presented hypertension, elevated levels of CRP, and insulin resistance. Furthermore, these studies reinforce the idea that classic risk factors explain only about 50–60% of CVD [39]; thus, there has been an increasing interest in identifying novel biomarkers that might improve the global risk prediction of CVD [40, 41].

This study aimed to evaluate the influence of low HDL-c and/or elevate TGs levels on the cardiometabolic profile of patients with cardiovascular risk factors but average LDL-c contents, using both traditional and new nontraditional (emergent) markers, including HDL subpopulations, oxidized LDL (Ox-LDL), and inflammatory and angiogenesis mediators.

## 2. Materials and Methods

### 2.1. Subjects and Ethical Consideration

One hundred and sixty nine patients with cardiovascular risk factors were enrolled in the study, divided in two major groups based on the HDL-c levels: a group of patients with normal HDL-c levels (including 119 subjects, 71 males, and 48 females) and another one with low HDL-c concentrations (of 50 patients, 17 males and 33 females), using the cutoffs of 1.03 mmol/L for men and 1.29 mmol/L for women. In addition, each group was subdivided into two subgroups on the basis of their TGs levels: a subgroup of patients with normal TGs levels and another one with high TGs contents, using the cutoff of < and ≥ of 1.69 mmol/L. Thus, four subgroups were analyzed: (A) normal HDL-c and normal TGs (*n* = 83), (B) normal HDL-c and high TGs (*n* = 36), (C) low HDL-c and normal TGs (*n* = 17), and (D) low HDL-c and high TGs (*n* = 33). The cutoffs for HDL-c and TGs levels were chosen according to the NCEP-ATP III guidelines. All groups were defined as having cardiovascular risk factors in terms of previous diagnosis and/or pharmacological treatment for hypertension and/or for type 2 diabetes mellitus (T2DM) and/or for dyslipidemia. T2DM was diagnosed in the Diabetes and Metabolic Diseases Unit from the Coimbra Hospital Centre (EPE), according to the European Guidelines. Patients presenting previous diagnosis and/or treatment for hypertension and dyslipidemia were recruited during the performance of routine laboratory analysis where they expressed taking antihypertensive therapy and/or lipid-lowering drugs after proper clinical and laboratorial diagnosis, which were performed according to the International Society of Hypertension/World Health Organization and the Seventh Joint National Committee on Hypertension and National Cholesterol Education Program-Adult Treatment Panel III (NCEP-ATP III) for hypertension and dyslipidemia, respectively. The patients from the groups of normal HDL-c and low HDL-c levels were under the following medication, respectively: (a) insulin and/or oral antidiabetic drugs (OAD): 57.98% and 68.00%; (b) lipid-lowering drugs: 65.55% (78 out of 119) in the normal HDL-c group, distributed by statins (58 patients), fibrates (15 patients), and a combination of both (5 patients) and 60.00% (30 out of 50) in the low HDL-c group, distributed by statins (21 patients), fibrates (6 patients), a combination of both (1 patient), ezetemibe (1 patient), and omega-3 (1 patient); (c) antihypertensive drugs: 68.07% and 76.00%. Pregnant women were excluded. The study was performed in agreement with the code of ethics of the World Medical Association (Declaration of Helsinki) for human studies and received authorization from the local ethics committee, as well as from all the participants by signing a written informed consent.

### 2.2. Data and Blood Collection

The following data was obtained from each subject by trained personnel: weight and height (without shoes and wearing light outdoor clothing) were measured in order to calculate body mass index (BMI), waist circumference (WC), and systolic and diastolic blood pressure (SBP and DBP), the latter of which were assessed in the sitting position after a 5 min rest. Blood samples were collected by venipuncture from the subjects after an overnight fasting period, via both EDTA-containing tubes and tubes without anticoagulant, in order to obtain plasma, buffy-coat, and serum and processed within 2 hours of collection. Aliquots were immediately stored at −80°C until assayed.

### 2.3. Assays

#### 2.3.1. Lipid and Glycemic Profiles

Serum total cholesterol (Total-c), HDL-c, LDL-c, and TGs were analysed on a Hitachi 717 analyser (Roche Diagnostics) using standard laboratorial methods. Total-c reagents and TGs kit were obtained from bioMérieux sa (Lyon, France). HDL-c Plus and LDL-c Plus tests were obtained from F. Hoffmann-La Roche Ltd (Roche Diagnostics Div., Basel, Switzerland). Plasma concentrations of Ox-LDL were evaluated by using a standard commercial enzyme-linked immunoassay (Oxidized LDL ELISA, Mercodia, Uppsala, Sweden). Serum glucose levels were measured using a Glucose Oxidase commercial kit (Sigma, St. Louis, MO, USA). HbA1c levels were analyzed on a Hitachi 717 analyser (Roche Diagnostics) using standard laboratory methods.

#### 2.3.2. HDL Subpopulations Assay

Subpopulations were separated and quantified using a Lipoprint kit from Quantimetrix Corp. (Redondo Beach, CA, USA). The assay involves a polyacrylamide gel electrophoresis assay and a complete Lipoprint System for data acquisition and quantification of large, intermediate, and small subpopulations of HDL. 

#### 2.3.3. PON1 Paraoxonase Activity

It was assessed spectrophotometrically and expressed in nmol of p-nitrophenol/mL/min. In brief, paraoxonase activity was measured by adding serum to 1 mL Tris/HCl buffer (100 mmol/L, pH 8.0) containing 2 mmol/L CaCl_2_ and 5.5 mmol/L paraoxon (O,O-diethyl-O-p-nitrophenylphosphate; Sigma Chemical Co). The rate of generation of p-nitrophenol was determined at 412 nm, 37°C, via the use of a continuously recording spectrophotometer (Beckman DU-68). 

### 2.4. Serum Inflammatory, Angiogenic, and Endothelial Markers

Serum adiponectin, tumor necrosis factor alpha (TNF-*α*), and vascular endothelial growth factor (VEGF) contents were assessed using Quantikine enzyme-linked immunoassays kits from R&D Systems (Minneapolis, USA); serum intercellular adhesion molecule 1 (iCAM1) levels were evaluated by using an Elisa kit from Abcam (Cambridge, MA, USA); high-sensitivity C-reactive protein (hsCRP) was evaluated by immunoturbidimetry, using commercially available kits (CRP [latex] High-Sensitivity, Roche Diagnostics); uric acid was analyzed on a Hitachi 717 analyser (Roche Diagnostics) using standard laboratory methods. 

### 2.5. Statistical Analysis

Statistical analysis was performed by using the IBM Statistical Package for Social Sciences (SPSS) for Windows, version 20.0, (SPSS, Inc., Chicago, IL, USA). The distribution of continuous variables was analyzed using Kolmogorov-Smirnov tests, to assess significant departures from normality. Comparisons between groups were performed using the Independent Samples *t*-test and the Mann-Whitney test. The association between categorical variables was analyzed using Pearson's test. Statistical significance was accepted at *P* less than 0.05.

## 3. Results

### 3.1. Anthropometric Data and General Characterization of Populations

One hundred and sixty nine patients were enrolled in the study: 119 with normal HDL-c values and 50 with low HDL-c contents. From the normal HDL-c group, the subgroup with normal TGs levels (population A) included 83 subjects and the subgroup with high TGs values (population B) included 36 individuals. Concerning the group with low HDL-c contents, 17 subjects were included in the subgroup with normal TGs contents (population C) and 33 individuals in the subgroup with high TGs levels (population D). Throughout the text, three main comparisons will be analyzed as a way to better dissect the differences and the effects of HDL-c and TGs levels variations on the distinct circumstances (as indicated in the tables): comparison 1: effects of TGs levels (normal versus high) on normal and low HDL-c conditions/populations, by comparing population A with B and C with D; comparison 2: effects of HDL-c levels (normal versus low) on normal and high TGs conditions/populations, by comparing population A with C and B with D; comparison 3: effects of variations of both HDL-c and TGs levels, by comparing population A with D and B with C. 

The demographic and anthropometric data of the 4 populations are summarized in Table 1. The normal and low HDL-c groups are matched concerning age, without changes between the 4 populations, while there were higher values of BMI and waist circumference in the subgroups with high TGs contents, independently of the HDL-c levels (populations B and D). The same populations also demonstrated a trend to higher glycemia contents, despite being not statistically significant. Concerning blood pressure, unchanged values were found between the subgroups in all the comparisons (Table 1). 

### 3.2. Classical Lipid Profile

**Figure 1:**
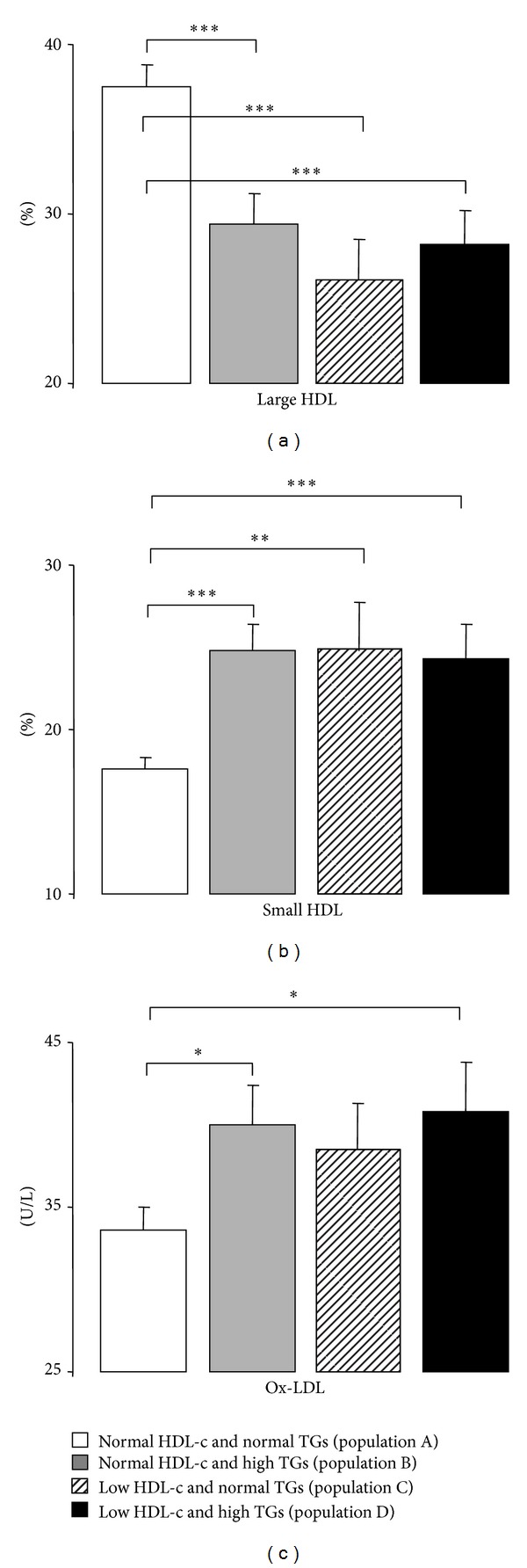
Serum Large HDL (a), small HDL (b), and Ox-LDL (c), in the study populations. Results are presented as mean ± SEM. *P < 0.05,**P < 0.01, and ***P < 0.001.

The majority of the patients were under antidyslipidemic therapy, which can justify some of the data obtained for the classic lipid profile. Concerning the first comparison (effects of TGs levels), the population B (with high TGs contents and normal HDL-c levels) presented, in comparison with the population A (with normal TGs and normal HDL-c levels), significantly higher values of all the lipidic parameters, including Total-c, TGs (as expected, by definition of groups), LDL-c, Ox-LDL, non-HDL-c, Total-c/HDL-c, LDL-c/HDL-c, and TGs/HDL-c (Table 2 and Figure 1(c)). Under conditions of low HDL-c, the subgroup with high TGs (population D) also presented significantly higher values of Total-c, TGs, non-HDL-c, Total-c/HDL-c, and TGs/HDL-c. 

Concerning the second comparison (effects of HDL-c levels), independently of the TGs contents (normal or high) the populations with low-HDL-c presented higher TGs, Total-c/HDL-c, LDL-c/HDL-c, and TGs/HDL-c, without changes on LDL-c, Ox-LDL, and non-HDL-c (Table 2 and Figure 1(c)).

Finally, the effects of simultaneous variations of TGs and HDL-c (comparison 3) were more pronounced when both parameters are out of average values: comparing the population A (of normal HDL-c and TGs) with the population D (of low HDL-c and high TGs); in fact, the population D presented significantly higher values of Total-c, TGs, LDL-c, Ox-LDL, non-HDL-c, Total-c/HDL-c, LDL-c/HDL-c, and TGs/HDL-c (Table 2 and Figure 1(c)). When comparing the population B (of normal HDL-c and high TGs) with the population C (of low HDL-c and normal TGs), the lipidic profile was clearly worse when TGs are higher than when HDL-c is low; in fact, population B presented significantly higher values of Total-c, TGs, non-HDL-c, LDL-c/HDL-c, and TGs/HDL-c (Table 2).

### 3.3. HDL Subpopulations and Paraoxonase Activity

Regarding the content of HDL subpopulations, Table 2 and Figures 1(a) and 1(b) express the effects of HDL-c and TGs levels. The lower values of HDL-c or the higher values of TGs both promoted the poor quality of HDL, viewed by significantly reduced percentage of large HDL subpopulations and increased percentage of small HDL ones. Paraoxonase activity was unchanged between the 4 populations under study in all the comparisons (Table 2).

### 3.4. Markers of Inflammation, Angiogenesis, and Endothelial Lesion

**Figure 2:**
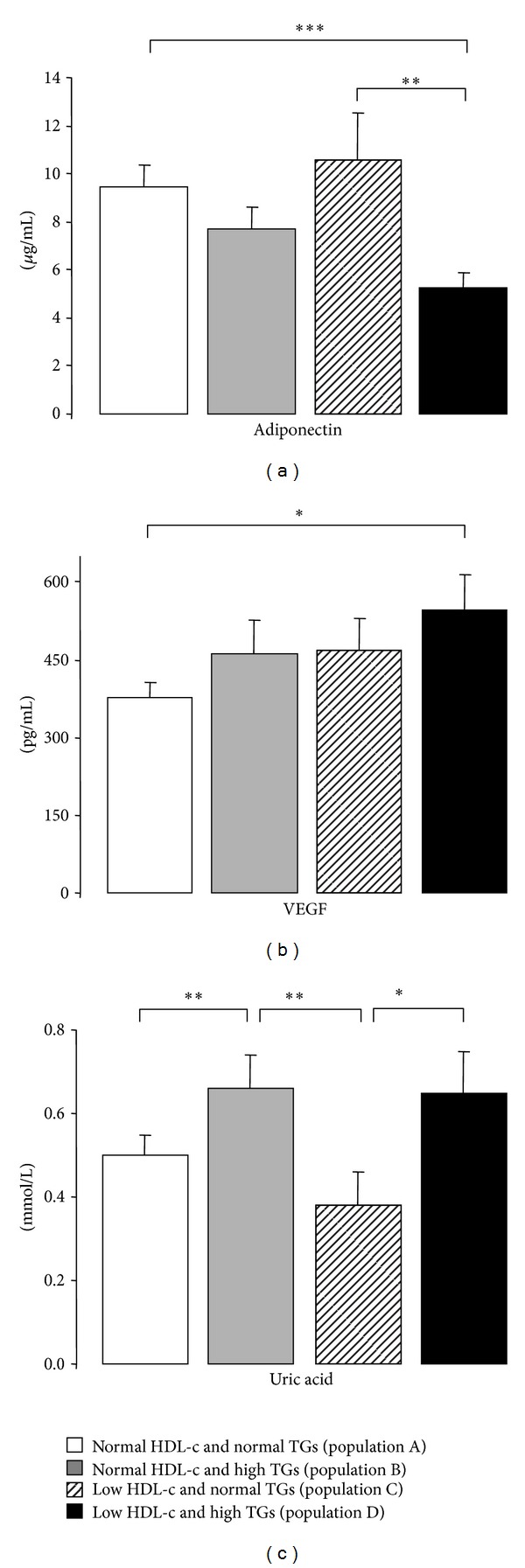
Serum adiponectin (a), VEGF (b), and uric acid (c) levels, in the study populations. Results are presented as mean ± SEM. *P < 0.05, **P < 0.01, and ***P < 0.001.

hsCRP, TNF-*α*, and iCAM-1 contents were unchanged between populations, despite a trend to reduce iCAM-1 in the population D (of low HDL-c and high TGs levels), which is significantly lower when compared with the population A (of normal HDL-c and normal TGs levels) (Table 3). Adiponectin concentration is significantly lower only in the subgroup with low HDL-c and high TGs (population D), and the value is statistically significant when compared with the population A (of normal HDL-c and normal TGs levels) and with population C (of low HDL-c and normal TGs levels) (Table 3 and Figure 2(a)). Similar profile was encountered for VEGF, with a significantly higher content in the subgroup with simultaneous change of HDL-c and TGs (population D), being statistically significant when compared with the subgroup with normal levels of both HDL-c and TGs (population A) (Table 3 and Figure 2(b)). Finally, uric acid values were higher in the subgroups with high TGs (population B versus A and D versus C), independently of normal or low HDL-c levels (Table 3 and Figure 2(c)).

### 3.5. Analysis of Correlations between Markers of CV Risk in All Study Subgroups

For some previously described markers, which presented changes between subgroups, there were significant correlations, particularly in the population A. The most interesting parameters in the correlation analysis were the large and small HDL subpopulations, Ox-LDL, adiponectin, uric acid, and waist circumference.

**Figure 3:**
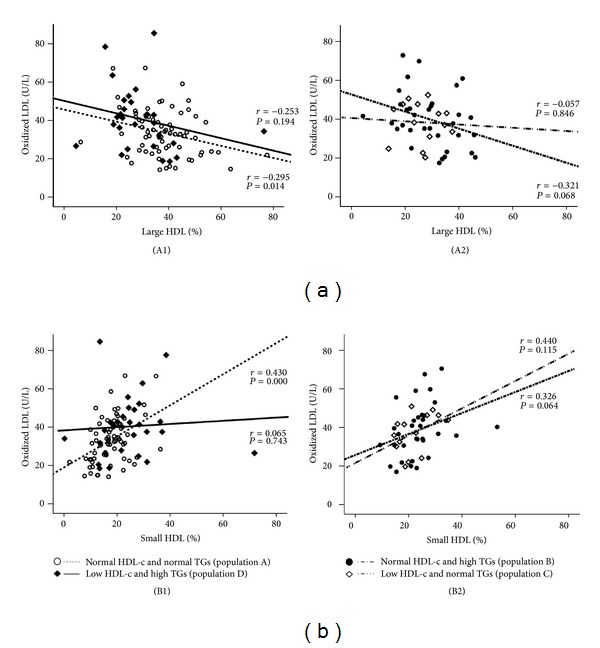
Correlations between Ox-LDL and large HDL (a) and small HDL (b) in the study populations. (A1) Population A: *r* = −0.295, *P* = 0.014; population D: *r* = −0.253, *P* = 0.194; (A2) population B: *r* = −0.321, *P* = 0.068; population C: *r* = −0.057, *P* = 0.846; (B1) population A: *r* = 0.430, *P* = 0.000; population D: *r* = 0.065, *P* = 0.743; (B2) population B: *r* = 0.326, *P* = 0.064; population C: *r* = 0.440, *P* = 0.115.

**Figure 4:**
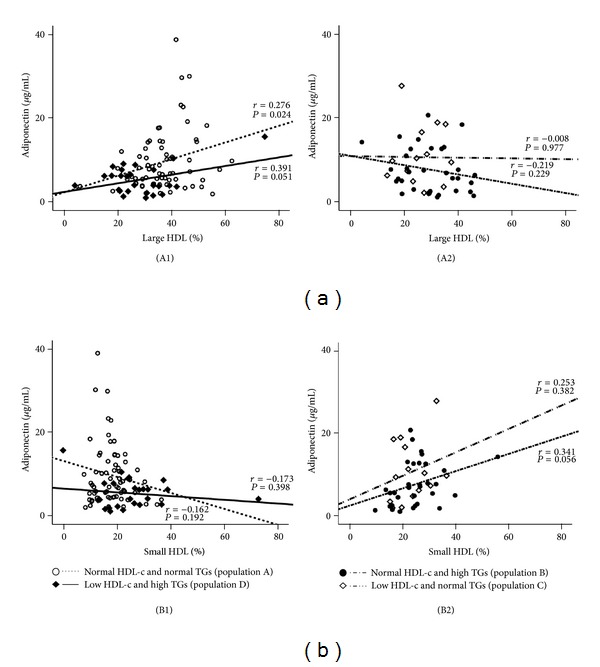
Correlations between adiponectin and large HDL (a) and small HDL (b) in the study populations. (A1) Population A: *r* = 0.276, *P* = 0.024; population D: *r* = 0.391, *P* = 0.051; (A2) population B: *r* = 0.219, *P* = 0.229; population C: *r* = −0.008, *P* = 0.977; (B1) population A: *r* = −0.162, *P* = 0.192; population D: *r* = −0.173, *P* = 0.398; (B2) population B: *r* = 0.341, *P* = 0.056; population C: *r* = 0.253, *P* = 0.382.

**Figure 5:**
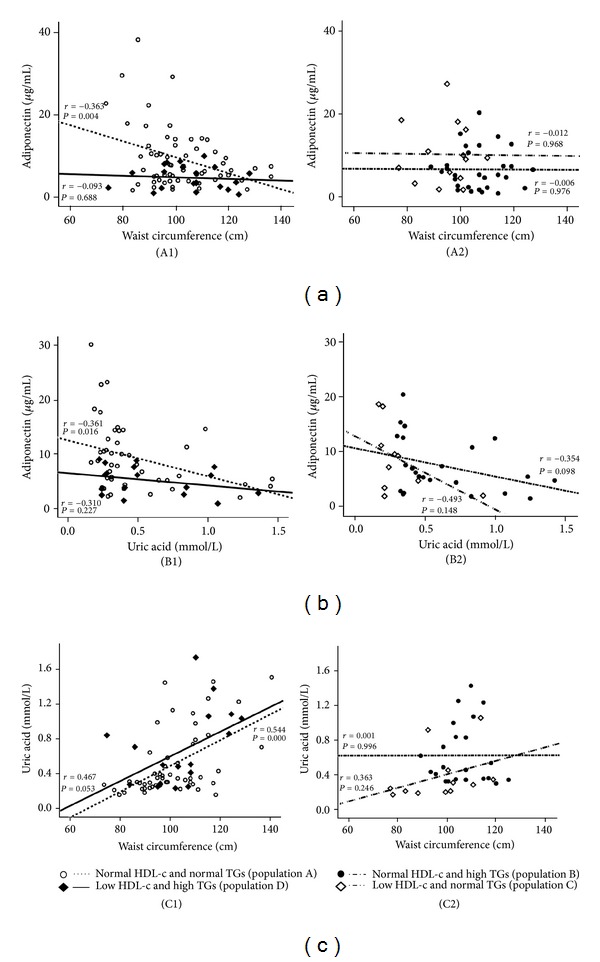
Correlations between adiponectin and waist circumference (a), adiponectin and uric acid (b), and uric acid and waist circumference (c) in the study populations. (A1) Population A: *r* = −0.363, *P* = 0.004; population D: *r* = −0.093, *P* = 0.688; (A2) population B: *r* = −0.006, *P* = 0.976; population C: *r* = −0.012, p = 0.968; (B1) population A: *r* = −0.361, *P* = 0.016; population D: *r* = −0.310, *P* = 0.227; (B2) population B: *r* = −0.354, *P* = 0.098; population C: *r* = −0.493, *P* = 0.148; (C1) population A: *r* = 0.544, p = 0.000; population D: *r* = 0.467, *P* = 0.053; (C2) population B: *r* = 0.001, *P* = 0.996; population C: *r* = 0.363, *P* = 0.246.

The values of Ox-LDL in the normal HDL-c and normal TGs subgroup (population A) were negatively and significantly correlated with large HDL (*r* = −0.295, *P* = 0.014) and positively and significantly correlated with small HDL (*r* = 0.430, *P* = 0.000); these correlations were not found in the 3 other populations (Figure 3). Concerning adiponectin, in the population A there was a significant positive correlation with large HDL (*r* = 0.276, *P* = 0.024) and a trend to a negative correlation with small HDL (*r* = −0.162, *P* = 0.192); these correlations were not found in the other 3 populations (Figure 4). Furthermore, also in the population A, adiponectin was negatively and significantly correlated with waist circumference (*r* = −0.363, *P* = 0.004) and uric acid (*r* = −0.361, *P* = 0.016), which also correlates positively and significantly waist circumference (*r* = 0.544, *P* = 0.000); once again, these correlations were absent (not statistically significant) in the other 3 populations (Figure 5). 

## 4. Discussion

It is now widely recognized that the current lipid-lowering therapies, in particular those directed to reduce LDL-c levels, such as statins, are insufficient to prevent part of the cardiovascular events; indeed, residual cardiovascular risk (RCVR) remains elevated even in clinical trials in which LDL-c levels have been aggressively reduced [11–13, 42–44]. As a result, there has been an increased focus on elevated TGs levels and low HDL-c levels and their significant contributions to RCVR even when LDL-c levels are well controlled [14, 15]. For example, Genest et al. reported that although 34% of patients with premature heart disease had LDL-c levels > 160 mg/dL, more than half of the patients with premature heart disease (57%) had low HDL-c levels [34, 45]. Additionally, it has been reported that, in both male and female patients with premature CAD, the greatest risk factor is actually low HDL-c levels, though these individuals often possess high TGs levels as well. Conversely, the study found that TGs levels were significantly higher and HDL-c levels were significantly lower in men and women with premature CAD, compared with patients from the Framingham Offspring Study who were free of CHD at baseline [14, 34]. 

Low levels of HDL-c and elevated levels of TGs have been largely recognized as risk factors for coronary heart and/or artery disease [16–18, 29–31], particularly in patients with both conditions [34, 35]. Thus, in statin-medicated patients, with average LDL-c levels, HDL-c and TGs have been gaining particular relevance, as good measures of RCVR and target for prevention of cardiovascular events. Several lines of evidence reinforce the idea that traditional risk factors, including lipidic (such as LDL-c), might not tell the whole story about CVD progression and prevention of CV events, and thus, other lipid fractions/components, such as HDL, TGs, and oxidized LDL (Ox-LDL), might have a major role as biomarkers and/or targets to the reduction of overall cardiovascular health [14, 15, 46, 47]. Actually, there has been an increasing interest in identifying novel biomarkers, including lipidic, inflammatory, and angiogenic, that might improve the global risk prediction of CVD [40, 41]. The present study aimed to evaluate the influence of low HDL-c and/or elevate TGs levels and the relative relevance of each one alone and combined on the cardiometabolic profile of patients with cardiovascular risk factors but average LDL-c contents, using both traditional and new nontraditional (emergent) markers, including HDL subpopulations, Ox-LDL, and inflammatory and angiogenesis mediators.

The main finding of this study is that patients with controlled LDL-c levels, as a result of antidyslipidemic therapy, in particular with statins, present a poor cardiometabolic profile as a result of isolate or combined low-HDL-c dyslipidemia and hypertriglyceridemia. This cardiometabolic profile, which might be viewed as a residual (but not negligible) cardiovascular risk, is better diagnosed when analyzed in terms of nontraditional markers, including large and small HDL subpopulations, Ox-LDL, adiponectin, VEGF, and uric acid.

Regarding the obesity profile, the results clearly show that patients with elevated TGs levels, regardless of the HDL-c values (normal or low concentrations), present the highest BMI (above 30 kg/m^2^) and waist circumference values. Several studies have linked obesity to high TGs and low HDL-c levels [48, 49]. In our study, elevated concentration of TGs is more important in determining obesity in patients with cardiovascular risk factors. This is better viewed when comparing patients with average TGs contents and normal HDL-c (population A) versus low HDL-c (population C) levels, showing unchanged values between the groups, despite both populations already presenting overweight (with BMI above 28 kg/m^2^ for both). Similar profile was encountered for Total-c with significantly higher levels in the populations with elevated TGs contents, regardless of HDL-c concentration. Concerning LDL-c and Ox-LDL, we found that the highest values are encountered in the populations with high TGs concentrations, despite a trend of increased contents in the population with low HDL-c values and normal TGs contents. Thus, once again, elevated concentration of TGs is more important than HDL-c in determining the values of LDL-c and of Ox-LDL, in patients with cardiovascular risk factors and under antidyslipidemic medication.

The association between cardiovascular disease and oxidation of LDL has been largely demonstrated and previous studies have reported an interesting relationship between Ox-LDL and markers of lipid profile, in populations with cardiovascular risk as well as in healthy individuals [27, 50]. One of the most relevant associations is related with inverse relationship and opposite roles of oxidized LDL and HDL-c on atherogenesis and CAD [47, 51–53]. Concerning the CAD, Ox-LDL is a promoter of key steps in the onset and evolution of atherosclerosis, including stimulation of monocyte infiltration and smooth muscle cell migration and proliferation; conversely, high levels of HDL-c prevent the development of atherosclerosis and CAD, in particular due to the transport of reserve cholesterol and the inhibition of Ox-LDL-induced monocyte infiltration; indeed, Ox-LDL and HDL-c are antagonists in the development of CVD [51]. Our previous studies, both in healthy individuals and in populations with cardiovascular risk, such type 2 diabetes patients, strongly suggested an association between Ox-LDL and HDL subpopulations, which was less evident with total HDL-c content [25, 26]. In fact, Ox-LDL concentrations showed an inverse and significant correlation with the large HDL subfractions, those viewed as more atheroprotective, and a direct and significant correlation with the small HDL subpopulations, which are indicated as the atherogenic ones, or at least less atheroprotective [25, 26]. Our present study reinforces these associations, since Ox-LDL levels presented the same significant correlation with the HDL subfractions: inverse with the large subpopulations and direct with the small HDL ones, in particular in the subgroup of patients with normal HDL-c and normal TGs values. Despite the recognition of an association between low levels of HDL-c with increased risk for CAD [52, 53], it has been suggested during the last years that a better indicator of HDL functionality may be their quality, which depends on its subpopulation's type (large versus small) and constituents, including PON1 activity [54–56]. In our study, HDL quality is reduced in the two populations with high TGs contents as well as in the population with normal TGs but low HDL-c concentration. Thus, both the low HDL-c dyslipidemia and the hypertriglyceridemia are, per se, even not cumulatively, promoters of poor HDL quality, viewed by the simultaneous reduced content of the large HDL subpopulations and increased of small ones. This profile was similar to that found for Ox-LDL concentration, which showed interesting correlations with the subpopulations, as previously reported. The implication of TGs levels in HDL quality has been gaining importance, as reported by other authors [57, 58]. These studies suggested that particle size of HDL subclasses tend to be small with increasing TGs concentration, indicating that HDL maturation might be hampered and efficiency of reverse cholesterol transport (RCT) might be weakened. These data suggest that TGs levels were not only significantly associated with, but liner with, the contents of HDL subfractions. Concerning the influence of HDL-c levels on HDL subpopulations, some studies have demonstrated that subjects with low HDL-c display marked changes in their HDL composition and subclass distribution; some of them indicate that the percentage of larger subfractions as well as HDL mean particle size is reduced in subjects with low HDL-c levels [59, 60], which is in agreement with our results. PON1 activity, which have been indicated as an indicator of HDL functionality, was unchanged between groups, which is in agreement with previous data from us in other populations of cardiovascular risk [25–27], suggesting that HDL subpopulations, rather than PON1 activity, are best markers of HDL quality. Whether HDL function and/or composition, rather than total HDL-c content, are better biomarkers of residual CV risk is an exciting (yet open) issue that deserves additional efforts from the scientific clinical community.

Inflammation and oxidative stress are key pathways for endothelial dysfunction and development of atherosclerosis, being oxidized LDL one of the major players in this process, together with several mediators of inflammation [46, 47, 61]. HDL exerts direct endothelial-protective effects, such as stimulation of endothelial production of the antiatherogenic molecule nitric oxide, as well as antioxidant, anti-inflammatory and antithrombotic effects [62–64]. An inflammatory imbalance, as manifested by increased proinflammatory cytokines, such as the tumor necrosis factor alpha (TNF-*α*), increased C-reactive protein (CRP), and/or reduced levels of anti-inflammatory and antiatherogenic mediators, such as adiponectin, has been considered a key factor for the increased cardiovascular risk in some pathologies [65, 66]. Similar importance is now attributed to the phenomenon of angiogenesis, which has the vascular endothelial growth factor (VEGF) as the key biomarker, a peptide growth factor secreted by vascular endothelial cells that stimulates vasculogenesis and angiogenesis, which has been involved in the pathogenesis of cardiovascular diseases, such as atherosclerosis [67], as well as to the intracellular adhesion molecules, such as ICAM-1, which has been associated with the severity of atherosclerosis and cardiovascular events [68]. Another new marker that deserves our attention is the uric acid; although uric acid can act as an antioxidant, excess serum accumulation is often associated with several conditions and has been suggested as an independent risk factor for carotid atherosclerosis in patients with CVD, such as in type 2 diabetes [69]. In our study, although unchanged values of high sensitivity CRP, TNF-*α* and iCAM-1 were found between the four populations under evaluation, a reduced concentration of adiponectin and an increased content of VEGF in the patients's population with low HDL-c and high TGs levels were encountered, suggesting that both conditions contribute to these alterations. Previous studies have indicated an association of lower adiponectin levels in populations with increased values of TGs and reduced values of HDL-c, as well as in healthy individuals [70, 71]. In our study, adiponectin levels showed important correlation with HDL subpopulations, inversely with small HDL subfractions and direct with large ones, but not with total HDL-c content; in addition, adiponectin levels also presented inverse correlation with waist circumference. Once again, those correlations were more evident and strong in the population with normal HDL-c and normal TGs contents, as occurred also for the correlations between Ox-LDL and HDL subpopulations. VEGF serum concentrations were previously correlated with parameters of lipid profile, including TGs, in hypercholesterolemic patients [72]. Finally, uric acid contents were significantly increased in both patients' populations with high TGs levels, regardless of HDL-c values, suggesting a direct impact of TGs, as previously reported [73]. Uric acid levels presented significant inverse correlation with adiponectin and direct with waist circumference, in particular in the population with normal HDL-c and normal TGs contents, in agreement with the previously reported correlations, between both Ox-LDL and adiponectin and the large and small HDL subpopulations, suggesting a strong relationship between these lipidic, oxidative and inflammatory factor, which might be described yet as non-traditional markers. The fact that these associations are less evident in the subgroups of patients with low HDL-c levels and/or high TGs contents seem to indicate that under HDL-c dyslipidemia and/or hypertriglyceridemia there is a deregulation of the factors (lipidic, oxidative, and inflammatory), with a putative important impact on the evolution of cardiometabolic vascular disease.

Considering the cardiometabolic impact of low-HDL-c and/or high TGs levels on this type of patients with previous cardiovascular risk factors, even when LDL-c concentrations are adequately managed by antidyslipidemic therapy, therapeutic measures able to improve HDL-c levels and their quality/functionality and to reduce TGs concentration might be of key importance to reduce the residual risk previously identified on this type of populations, namely, by reducing the oxidative, inflammatory and angiogenic mechanisms underlying the evolution of disease. Since the current therapeutic arsenal is of limited impact on HDL-c levels, in particular the most popular medication, such as statins, and since a percentage of patients (of concern) present low HDL-c dyslipidemia and/or hypertriglyceridemia, nonpharmacological measures (including regular physical exercise and low-fat and low-sugar diets) might deserve more attention, as well as new and more effective agents that might prove efficacy to reduce TGs concentration and improve HDL quality and their beneficial effects, including reduction of Ox-LDL as well as of deleterious inflammatory mediators. Despite some disappointing results of some of the recent clinical trials aimed to access the putative benefits of pharmacotherapy targeting HDL cholesterol, particularly in terms of side-effects and short and long-term outcome data of cardiovascular events [74], there are positive results related to the impact of dalcetrapib, a cholesteryl ester transfer protein (CETP) inhibitor, on HDL quality [75]. This apparent discrepancy raises the question as to whether certain biomarkers are relevant either in specific patient populations or on the background of therapies, such as statins. More clinical data of large clinical trials designed to evaluate the impact on cardiovascular events of drugs directed to modulate HDL concentration and/or quality are needed to better elucidate this issue.

## 5. Conclusions

In a patient population with cardiovascular risk factors, low HDL-c and/or high TGs levels are associated with a poor cardiometabolic profile. This condition that often occurs in patients under lipid-lowering therapy with average LDL-c concentrations has been called residual cardiovascular risk, and the patients are those that frequently experience nonfatal and fatal cardio- and cerebrovascular events. Our study suggests that this residual cardiovascular risk is better viewed by nontraditional (emergent) lipid biomarkers, including HDL subpopulations, oxidized LDL, as well as markers of inflammation and angiogenesis, such as adiponectin, uric acid, and VEGF. Proper pharmacological and nonpharmacological therapeutic interventions directed to raise HDL-c levels and functionality and to reduce TGs levels are advisory preventive measures in this type of CV risk populations.

## Figures and Tables

**Table 1:** Anthropometric data and general characterization of the populations under study.

Parameters	(1) Effects of TGs levels (normal versus high) on normal and low HDL-c conditions/populations						(2) Effects of HDL-c levels (normal versus high) on normal and high TGs conditions		(3) Effects of variations of both HDL-c and TGs levels	
	Normal HDL-c (*n* = 119)			Low HDL-c (*n* = 50)						
	Normal TGs (*n* = 83) Population A	High TGs (*n* = 36) Population B	*P* A versus B	Normal TGs (*n* = 17) Population C	High TGs (*n* = 33) Population D	*P* C versus D	(Normal TGs) *P* (A versus C)	(High TGs) *P* (B versus D)	*P* A versus D	*P* B versus C
Age (years)	60.12 ± 1.06	58.69 ± 1.78	0.491	61.88 ± 2.76	60.42 ± 1.36	0.640	0.508	0.449	0.873	0.324
BMI (Kg/m^2^)	28.52 ± 0.52	30.73 ± 0.77	0.020	28.27 ± 0.85	30.69 ± 0.81	0.046	0.839	0.972	0.027	0.048
WC (cm)	102.16 ± 1.60	106.61 ± 1.75	0.049	97.71 ± 2.78	105.42 ± 2.74	0.045	0.228	0.707	0.306	0.007
SBP (mmHg)	140.64 ± 2.68	139.18 ± 3.10	0.748	136.12 ± 5.83	134.33 ± 3.58	0.785	0.485	0.259	0.191	0.939
DBP (mmHg)	77.85 ± 1.42	78.40 ± 2.19	0.830	74.82 ± 3.62	75.67 ± 2.27	0.838	0.392	0.389	0.414	0.535
Glycemia (mmol/L)	7.88 ± 0.41	8.92 ± 0.64	0.096	7.62 ± 0.80	9.94 ± 0.76	0.064	0.759	0.404	0.015	0.084
HbA1c (%)	7.90 ± 0.24	8.61 ± 0.47	0.143	8.42 ± 0.75	9.75 ± 0.44	0.126	0.431	0.082	0.000	0.833

Results are presented as media ± SEM. Independent samples *t*-test and Mann-Whitney test for normalized and nonnormalized samples, respectively. BMI: body mass index; HbA1c: glycated hemoglobin; SBP: systolic blood pressure; DBP: diastolic blood pressure; WC: waist circumference.

**Table 2:** Markers of lipid profile of the populations under study.

Parameters	(1) Effects of TGs levels (normal versus high) on normal and low HDL-c conditions/populations						(2) Effects of HDL-c levels (normal versus high) on normal and high TGs conditions		(3) Effects of variations of both HDL-c and TGs levels	
	Normal HDL-c (*n* = 119)			Low HDL-c (*n* = 50)						
	Normal TGs (*n* = 83) Population A	High TGs (*n* = 36) Population B	*P* A versus B	Normal TGs (*n* = 17) Population C	High TGs (*n* = 33) Population D	*P* C versus D	(Normal TGs) *P* (A versus C)	(High TGs) *P* (B versus D)	*P* A versus D	*P* B versus C
Total-c (mmol/L)	4.60 ± 0.10	5.54 ± 0.20	0.000	4.48 ± 0.19	5.45 ± 0.20	0.004	0.599	0.768	0.000	0.002
TGs (mmol/L)	1.06 ± 0.03	2.53 ± 0.13	0.000	1.23 ± 0.07	3.23 ± 0.19	0.000	0.049	0.006	0.000	0.000
HDL-c (mmol/L)	1.50 ± 0.03	1.42 ± 0.04	0.229	1.04 ± 0.04	1.00 ± 0.03	0.385	0.000	0.000	0.000	0.000
Large HDL (%)	37.54 ± 1.25	29.41 ± 1.83	0.000	26.10 ± 2.38	28.16 ± 2.06	0.541	0.000	0.646	0.000	0.295
Interm HDL (%)	44.83 ± 0.72	45.74 ± 0.97	0.474	48.95 ± 1.36	47.42 ± 1.34	0.667	0.017	0.311	0.071	0.064
Small HDL (%)	17.62 ± 0.72	24.84 ± 1.57	0.000	24.92 ± 2.99	24.32 ± 2.06	0.937	0.005	0.991	0.000	0.927
LDL-c (mmol/L)	2.61 ± 0.09	3.02 ± 0.19	0.049	2.89 ± 0.18	3.09 ± 0.20	0.517	0.176	0.831	0.029	0.937
Ox-LDL (U/L)	33.65 ± 1.40	40.00 ± 2.43	0.018	38.55 ± 2.77	40.83 ± 3.01	0.825	0.148	0.830	0.016	0.728
Ox-LDL/LDL-c	12.85 ± 0.39	13.73 ± 0.68	0.229	13.34 ± 1.02	14.33 ± 0.84	0.476	0.616	0.572	0.153	0.655
Non-HDL-c (mmol/L)	3.10 ± 0.09	4.12 ± 0.21	0.000	3.44 ± 0.18	4.45 ± 0.19	0.002	0.111	0.242	0.000	0.047
Total-c/HDL-c	3.17 ± 0.08	4.03 ± 0.19	0.000	4.40 ± 0.25	5.56 ± 0.23	0.002	0.000	0.000	0.000	0.194
LDL-c/HDL-c	1.82 ± 0.08	2.22 ± 0.16	0.037	2.84 ± 0.23	3.15 ± 0.23	0.539	0.000	0.001	0.000	0.032
TGs/HDL-c (mmol/L)	0.75 ± 0.03	1.85 ± 0.12	0.000	1.20 ± 0.09	3.38 ± 0.25	0.000	0.000	0.000	0.000	0.000
PON1 activity	487.23 ± 16.04	509.12 ± 37.36	0.593	523.43 ± 64.79	503.52 ± 39.36	0.778	0.776	0.918	0.902	0.810

Results are presented as media ± SEM. Independent samples *t*-test and Mann-Whitney test for normalized and nonnormalized samples, respectively. HDL-c: high-density lipoprotein cholesterol; LDL-c: low-density lipoprotein cholesterol; Ox-LDL: oxidized low-density lipoprotein; TGs: triglycerides; Total-c: total cholesterol.

**Table 3:** Markers of inflammatory and angiogenic profile of the populations under study.

Parameters	(1) Effects of TGs levels (normal versus high) on normal and low HDL-c conditions/populations						(2) Effects of HDL-c levels (normal versus high) on normal and high TGs conditions		(3) Effects of variations of both HDL-c and TGs levels	
	Normal HDL-c (*n* = 119)			Low HDL-c (*n* = 50)						
	Normal TGs (*n* = 83) Population A	High TGs (*n* = 36) Population B	*P* A versus B	Normal TGs (*n* = 17) Population C	High TGs (*n* = 33) Population D	*P* C versus D	(Normal TGs) *P* (A versus C)	(High TGs) *P* (B versus D)	*P* A versus D	*P* B versus C
CRP (*µ*g/mL)	0.42 ± 0.09	0.35 ± 0.08	0.798	0.59 ± 0.16	0.60 ± 0.12	0.981	0.206	0.237	0.131	0.327
TNF-*α* (pg/mL)	3.21 ± 0.36	2.96 ± 0.39	0.727	2.83 ± 0.43	3.50 ± 0.55	0.758	0.914	0.552	0.573	0.858
iCAM-1 (ng/mL)	580.98 ± 32.51	553.40 ± 61.34	0.664	536.10 ± 55.09	426.54 ± 26.89	0.064	0.525	0.215	0.001	0.975
Adiponectin (*µ*g/mL)	9.48 ± 0.87	7.67 ± 0.93	0.108	10.55 ± 1.96	5.22 ± 0.66	0.010	0.697	0.110	0.000	0.201
Uric acid (mmol/L)	0.50 ± 0.05	0.66 ± 0.08	0.005	0.38 ± 0.08	0.65 ± 0.10	0.023	0.104	0.616	0.144	0.005
VEGF (pg/mL)	376.48 ± 30.81	461.24 ± 63.74	0.161	468.45 ± 61.39	546.34 ± 67.86	0.759	0.113	0.302	0.017	0.564

Results are presented as media ± SEM. Independent samples *t*-test and Mann-Whitney test for normalized and nonnormalized samples, respectively. CRP: C-reactive protein; iCAM-1: intercellular adhesion molecule 1; TNF-*α*: tumour necrosis factor alpha; VEGF: vascular endothelial growth factor.
